# The Janus of Oxidative Stress Signaling in Different Pathophysiological Conditions

**DOI:** 10.1155/2013/708796

**Published:** 2013-06-19

**Authors:** Sumitra Miriyala, Manikandan Panchatcharam, Aimee Landar, Meera Ramanujam, Saurabh Chatterjee, Anantharaman Muthuswamy

**Affiliations:** ^1^Department of Cellular Biology and Anatomy, LSU Health Sciences Center Shreveport, LA 71103, USA; ^2^Department of Pathology, University of Alabama at Birmingham, AL 35233, USA; ^3^Immunology and Inflammation, Boehringer Ingelheim Pharmaceuticals, Inc., Ridgefield, CT 06877, USA; ^4^Environmental Health and Disease Laboratory, University of South Carolina, Columbia, NC 27709, USA; ^5^National Primate Research Center, University of Wisconsin-Madison, Madison, WI 53715, USA

While reactive oxygen species (ROS) are important for normal cellular activities, deviant production of ROS, or diminished capacity to scavenge excessive ROS, leads to an imbalance in the redox environment of the cell. Because of the dual role of ROS in cells in the production and removal of cellular ROS, a greater understanding of oxidative stress, under both normal and disease-causing conditions, and the involvement of cell organelle (mitochondrial, endoplasmic reticulum) ROS in global regulation of gene expression can illuminate the contribution of mitochondria and other cell organelles in the development of disease and may lead to the advancement of new and novel therapeutic modalities that exploit oxidative stress in treating many diseases.

Understanding the role of ROS signaling and redox biology in pathophysiological conditions is reflected by the wised range of topics covered in this special issue. 

T. Peng et al. underscore dual phase of mitochondrial respiratory chain defective cells harboring less mitochondrial stress due to low mitochondrial respiratory chain activity during mitochondrial ROS-mediated mitochondrial Ca^2+^ stress during severe oxidative insult.

N. V. Gorbunov et al. propose that the cell survival mechanisms activated in lipopolysaccharide-treated mesenchymal stromal cells in vitro could be a part of adaptive responses employed by stromal cells under septic conditions.

F. Tseng et al. provided a platform for an in vitro assay to characterize the effects of bone marrow mesenchymal stem cells on lipopolysaccharide-stimulated microglia. A powerful cell culture tool for investigating the molecular and cellular changes in microglia are bone marrow mesenchymal stem cells cocultures.

R. Dumitrascu et al. have shown that obstructive sleep apnea is an independent risk factor for cardiovascular disease such as arterial hypertension, heart failure, and stroke. The results clearly show that radical flux exerts direct cytotoxic effects, decreases NO bioavailability, enhances lipid peroxidation, increases sympathetic activity, and activates the proinflammatory transcription factor NF-*κ*B leading to the well-known clinical manifestations of obstructive sleep apnea in the cardiovascular disease system.

C. Tronel et al. demonstrated the involvement of Fe^2+^ in brain ROS production and the deleterious effects of heme-oxygenase-1 expression in vivo neuroinflammatory model linked to a hyperproduction of ROS, itself promoted by Fe^2+^ liberation.

M. Godínez-Rubí et al. have reviewed on the role of nitric oxide donors as possible neuroprotective therapeutic agents for ischemia/reperfusion treatment.

S. Whelan and B. S. Zuckerbraun manuscript reviews on the mitochondria signaling to other components of stress response via ROS, the unfolded protein response, mitochondrial autophagy, and biogenesis. The avenues of mitochondrial signaling were discussed in this review.

Y. Zhou et al. discuss how ROS regulates different steps in vascular development, including smooth muscle cell differentiation, angiogenesis, endothelial progenitor cells recruitment, and vascular cell migration, while Y. J. H. J. Taverne et al. review focuses on the function of ROS in cardiovascular pathology and on the effects of antioxidants on cardiovascular outcomes with emphasis on the so-called oxidative paradox.

A. J. Lepedda et al. suggest the presence of a more pronounced oxidative environment in unstable plaques. Identifying specific oxidative modifications and understanding their effects on protein function could provide further insight into the relevance of oxidative stress in atherosclerosis.

E. Menshchikova et al. data appear to indicate a possible role of hydrogen peroxide in intercellular communication during organization, maturation, and “dissociation” of granulomas in the dynamics of the process.

X. Zhan et al. study eventually addresses the mechanisms and biological functions of tyrosine nitration in pituitary tumorigenesis and will discover nitro protein biomarkers for pituitary adenomas and targets for drug design for pituitary adenoma therapy.

A. V. Ermakov et al. provided in their in vitro data suggesting that the oxidized DNA is a stress signal released in response to oxidative stress in the cultured cells, and, possibly, in the human body; in particular it might contribute to systemic abscopal effects of localized irradiation treatments.

M. Tsai et al. study shows that enhanced prostacyclin synthesis reduced glial activation and ameliorated motor dysfunction in hemiparkinsonian rats. Prostacyclin may have a neuroprotective role in modulating the inflammatory response in degenerating nigrastriatal pathway.

J. Espino et al. underlie the antioxidant and immune enhancing actions displayed by melatonin, thereby providing evidence for the potential application of this indoleamine as a “replacement therapy” to limit or reverse some of the effects of the changes that occur during immunosenescence.

M. Jerkic et al. indicate that eNOS-derived ROS contributes to endothelial dysfunction and likely predisposes to disease manifestations in several organs of hereditary hemorrhagic telangiectasia patients.

B. Song et al. review aims are to briefly describe the mechanisms, functional consequences, and detection methods of mitochondrial dysfunction. They describe the advantages and limitations of the Cys-targeted redox proteomics method with alternative approaches. Finally, they discuss various applications of this method in studying oxidatively modified mitochondrial proteins in extrahepatic tissues or different subcellular organelles and translational research.

K. E. Al-Otaibiet et al. results suggest a significant role of oxidative stress, proinflammatory myeloperoxidase, and vasoregulatory nitric oxide in the pathogenesis of contrast-induced nephropathy. 

G. Aliev et al. provide a review discussing the link between cancer and Alzheimer disease via oxidative stress induced by nitric oxide-dependent mitochondrial DNA over proliferation and deletion.

S. Miriyala et al. provided a therapeutic approach showing that a novel tetra peptide derivative exhibits in vitro inhibition of neutrophil-derived reactive oxygen species and lysosomal enzymes release. 

A. S. Kunt and M. H. Andac have shown a clinical study proving that persistent oxidative stress during reperfusion may lead to depressed myocardial function resulting in low cardiac output syndrome necessitating inotropic or intra-aortic balloon counterpulsation support. Besides total antioxidant capacity decreases during operation in a significant proportion of patients undergoing isolated coronary artery bypass which is more prominent and serious. 

These manuscripts represent an exciting and insightful snapshot of current oxidative stress biology. State of the art, existing challenges and emerging future topics are highlighted in this special issue, which may inspire the reader and help advance the present redox biology.

